# Associations Between E-Cigarette Type, Frequency of Use, and Quitting Smoking: Findings From a Longitudinal Online Panel Survey in Great Britain

**DOI:** 10.1093/ntr/ntv078

**Published:** 2015-04-20

**Authors:** Sara C. Hitchman, Leonie S. Brose, Jamie Brown, Debbie Robson, Ann McNeill

**Affiliations:** ^1^Department of Addictions, Institute of Psychiatry, Psychology and Neuroscience, King’s College London, London, United Kingdom;; ^2^UK Centre for Tobacco and Alcohol Studies, United Kingdom;; ^3^Health Behaviour Research Centre, University College London, London, United Kingdom

## Abstract

**Introduction:**

E-cigarettes can be categorized into two basic types, (1) cigalikes, that are disposable or use pre-filled cartridges and (2) tanks, that can be refilled with liquids. The aims of this study were to examine: (1) predictors of using the two e-cigarette types, and (2) the association between type used, frequency of use (daily vs. non-daily vs. no use), and quitting.

**Methods:**

Online longitudinal survey of smokers in Great Britain was first conducted in November 2012. Of 4064 respondents meeting inclusion criteria at baseline, this study included (*N* = 1643) current smokers followed-up 1 year later. Type and frequency of e-cigarette use were measured at follow-up.

**Results:**

At follow-up, 64% reported no e-cigarette use, 27% used cigalikes, and 9% used tanks. Among e-cigarette users at follow-up, respondents most likely to use tanks versus cigalikes included: 40–54 versus 18–24 year olds and those with low versus moderate/high education. Compared to no e-cigarette use at follow-up, non-daily cigalike users were less likely to have quit smoking since baseline (*P* = .0002), daily cigalike or non-daily tank users were no more or less likely to have quit (*P* = .3644 and *P* = .4216, respectively), and daily tank users were more likely to have quit (*P* = .0012).

**Conclusions:**

Whether e-cigarette use is associated with quitting depends on type and frequency of use. Compared with respondents not using e-cigarettes, daily tank users were more likely, and non-daily cigalike users were less likely, to have quit. Tanks were more likely to be used by older respondents and respondents with lower education.

## Introduction

There are an estimated 2.1 million e-cigarette users in Great Britain.^1^ Among smokers, 50.6% have ever used e-cigarettes, 17.6% currently use e-cigarettes, current use among former smokers is lower, estimated at 4.5%, and use among nonsmoking adults is estimated at 0.2%.^1^

E-cigarettes use battery power to heat an element to disperse a solution that usually contains nicotine.^2^ The dispersion of the solution leads to the creation of an aerosol that can be inhaled by the user. The heated solution typically contains propylene glycol or glycerine, water, nicotine, and flavorings. E-cigarettes do not contain tobacco, do not create smoke and do not rely on combustion. Consequently, they can deliver nicotine to the user with lower exposure to the harmful constituents that are produced by combustion of ordinary tobacco cigarettes.^3^ Several concerns and possible benefits of e-cigarettes have been discussed in the public health community. Concerns include use by youth, renormalization of smoking, deterring smoking cessation, and adverse health effects; while there is optimism about their use as a smoking cessation aid, and their potential to serve as a less harmful nicotine product for smokers.^4^

There is substantial heterogeneity between different types of e-cigarettes and the speed with which they are evolving making them difficult to categorize. E-cigarettes available in the United Kingdom can be classified into two basic types: (1) “cigalikes,” often resembling tobacco cigarettes, both disposable or with pre-filled cartridges and (2) “tanks,” designed to be refilled with liquid.^2,5^ In the United Kingdom, many of the most widely sold brands of cigalikes are now owned by the tobacco industry.^6^ To the authors’ knowledge, only one tobacco company owned e-cigarette company currently sells a tank model that is designed to be refilled with liquids by the user in the United Kingdom^7^; of note is that this company does not currently sell tanks in the United States that the authors are aware of.

Studies have validated the ability of e-cigarettes to deliver nicotine to the user. Blood plasma nicotine concentrations increase after inhalation of e-cigarette aerosol^5,8^ and cotinine, a biomarker for nicotine, has been detected in the saliva of e-cigarette users.^9,10^ The dose and rate at which an e-cigarette delivers nicotine to the user under fixed conditions, specifically, the nicotine per puff second, has been referred to as “nicotine flux.”^11,12^ Differences have also been found in the nicotine content of the aerosol within and across e-cigarette brands and liquids.^13–15^ There is also evidence that nicotine delivery differs by user, with more experienced users being able to obtain greater nicotine delivery, sometimes equivalent to ordinary tobacco cigarettes.^8,16,17^ This variation in user behavior has recently been attributed to puff duration, with longer puff duration leading to greater nicotine delivery.^18^ In addition to nicotine, differences have been found in the levels of carbonyl compounds in e-cigarette aerosol, such as formaldehyde.^19^ Tanks with variable voltage have also been shown to produce an aerosol with a greater concentration of carbonyl compounds when used at a higher voltage.^20^ However, recent commentary has raised problems with these studies, including that higher voltage levels would be aversive/unpalatable to users in real conditions.^21^

Because e-cigarettes have the ability to deliver nicotine there is a potential mechanism for e-cigarettes to function as a quitting aid.^22^ However, depending on the “nicotine flux” of an e-cigarette, some e-cigarette products and brands may be less effective than others for quitting, such that too little nicotine flux may not provide sufficient nicotine replacement for a smoker to quit, and too much may be unappealing if it causes side effects, such as nausea.^11,12^ One study comparing nicotine delivery among e-cigarette product types found that a popular tank model delivered more nicotine than a popular brand of cigalike.^23^ Another study found that inexperienced e-cigarette users rated cigalikes as less satisfying, but found no differences between cigalikes and tanks in relieving urges to smoke or withdrawal symptoms.^24^ If cigalikes deliver less nicotine than tanks given a fixed-type of user behavior, and are less satisfying to users in other ways, it is possible that they may be less effective for promoting smoking cessation.

Research also suggests that the types of e-cigarette products used by smokers and ex-smokers differ. Among a cross-sectional sample of 19 000 e-cigarette users from multiple countries,^25^ e-cigarette users who used cigalikes were less likely to be former smokers than those using tanks. Additionally, cross-sectional data show that among e-cigarette users, ex-smokers were more likely than current smokers to use tanks (54% vs. 35%).^1^

Studies using longitudinal data have examined the association between e-cigarette use and smoking cessation. However, to the authors’ knowledge, none of these studies have taken into account type of e-cigarette used, and only two have considered frequency of use. A study from Australia, Canada, United States, and United Kingdom found that e-cigarette users at follow-up were more likely to have reduced their cigarette consumption from 1 year earlier, but were not more likely to have quit.^26^ A web-based US study found that e-cigarette use at baseline did not prospectively predict smoking cessation 1 year later or lead to a reduction in cigarette consumption.^27^ Neither of the above studies considered frequency of use or type of e-cigarette. A longitudinal survey of established e-cigarette users, recruited from online e-cigarette user communities and smoking cessation websites, found that e-cigarette use was associated with reduced cigarette consumption.^28^ The two studies that have considered frequency of e-cigarette use have found differences in smoking behavior between more and less frequent users. Brose et al.^29^ found that respondents who used e-cigarettes daily at baseline were more likely to have made a quit attempt 1 year later, but were no more or less likely than those who did not use e-cigarettes to quit; daily e-cigarette use at follow-up was also associated with reduced cigarette consumption since baseline; no effects of non-daily e-cigarette use on quit attempts, quitting, or cigarette consumption were found. A study of 695 smokers from the United States found that those who reported using e-cigarettes daily for at least a month were more likely to have quit at follow-up, compared no use.^30^

Other studies have also examined the association between e-cigarette use and smoking cessation. In a cross-sectional study of 5863 English smokers who attempted to quit in the past year without using professional support, Brown et al.^31^ found that respondents who used e-cigarettes on their last quit attempt compared to over-the-counter nicotine replacement therapy or no quit aid were more likely to report abstinence.^31^ Two randomized control trials have also tested the efficacy of cigalike type e-cigarettes for smoking cessation. Bullen et al.^32^ found that e-cigarettes with or without nicotine were comparable to nicotine replacement therapy patches in supporting abstinence among smokers wanting to quit. Caponnetto et al.^33^ compared use of e-cigarettes among three groups of smokers not intending to quit (*N* = 300): with nicotine (7.2mg), with nicotine (7.2mg) but reducing nicotine over the study period (5.4mg), and nicotine-free. Caponnetto et al.^33^ found no differences among the three groups, with all groups showing similar declines in cigarette consumption and quitting, with an overall abstinence rate of 8.7% at 52 weeks.

To fill gaps in the current research on e-cigarettes and smoking cessation, this study aimed to investigate (1) baseline predictors of using cigalikes versus tanks among e-cigarette users at a 1-year follow-up and (2) the cross-sectional association between type of e-cigarette used, frequency of e-cigarette use, or no e-cigarette use at all and smoking cessation at follow-up, adjusting for baseline characteristics. Information on e-cigarette type was only collected at follow-up so it was not possible to examine the association with baseline data. These research questions were investigated using a web-based longitudinal (2012–2013) sample of adult smokers at baseline in Great Britain drawn from the general population. The baseline sample was previously described in a publication by Brown et al.^34^

## Methods

### Design

This study drew respondents from a longitudinal sample of smokers and ex-smokers recruited from an online panel managed by Ipsos MORI. Members of the online panel were invited by email to participate in a survey about smoking. Compensation for participation included points that could be used for high street vouchers or entry into a draw for prizes. Between November and December 2012, 23 785 respondents accepted the invitation and were asked a screening question about their smoking status of whom 25.9% (*n* = 6165) qualified for because they smoked in the past year; quotas were imposed to ensure broad representativeness by sex, age, and region. The proportion of past-year smoking was similar to a nationally representative sample of English smokers from a face-to-face survey.^35^ Of the 6165 respondents who met the inclusion criteria, *N* = 5000 fully completed the baseline survey. The follow-up survey was conducted 1 year later in December 2013 with an overall follow-up rate for the entire sample of 43.6% (*n* = 2182). The present study included current smokers only at baseline (*n* = 4064), of whom (*n* = 1759), were successfully followed-up (43.3%). Respondents who did not know their smoking status or reported they were exclusive users of other types of tobacco at follow-up were excluded in the final analysis, along with respondents with missing data or don’t know responses on key variables (*n* = 116), resulting in a final sample size of (*n* = 1643).

### Measures

#### Demographics and Smoking Behavior

##### Demographics at Baseline.

Measures included gender (male vs. female), age (18–24, 25–39, 40–54, and 55+), highest level of education, and annual household income. Education was categorized as: low = primary or secondary school/vocational level 1 & 2/trade apprenticeship, secondary school advanced/vocational level 3; moderate = further education/training college below degree level, or some university; and high = at least a university degree. Income was categorized as: low = under £30 000, moderate = £30 000–£44 999, high ≥ £45 000, and “no answer” (due to a high number of don’t knows/prefer not to say responses).

##### Smoking Status at Baseline and Follow-up.

Respondents were asked, “Which of the following best applies to you?” I smoke cigarettes (including hand-rolled) every day, I smoke cigarettes (including hand-rolled) but not every day, I do not smoke cigarettes at all but I do smoke tobacco of some kind (eg, pipe or cigar), I have stopped smoking completely in the last year, I stopped smoking completely more than a year ago, or don’t know/couldn’t say. Respondents who said at baseline they currently smoked cigarettes daily or non-daily were included in this study. At follow-up, ex-smokers were defined as having stopped smoking.

##### Strength of Urges to Smoke at Baseline.

Strength of urges to smoke (SUTS) at baseline was used as a measure of cigarette dependence; SUTS has been demonstrated to be a reliable predictor of short-term quitting among English smokers.^36^ Respondents were asked: “How much of the time have you felt the urge to smoke in the past 24 hours?” with responses: not at all, a little of the time, some of the time, a lot of the time, almost all of the time, all of the time, or don’t know. Respondents who felt urges to smoke were then asked: “In general, how strong have the urges to smoke been?” with responses: slight, moderate, strong, very strong, extremely strong, or don’t know. “Not at all” responses to the first question were coded as 0, and responses from the second question made up the remainder of the scale leading to a final measure of 0 (no urges) to 5 (extremely strong urges). Don’t know responses were excluded.

##### Motivation to Stop Smoking at Baseline.

Motivation to stop smoking at baseline (MTSS) has been shown to predict quit attempts among English smokers.^37^ Smokers were asked: “Which of the following best describes you?” with responses: I REALLY want to stop smoking and intend to in the next month, I REALLY want to stop smoking and intend to in the next 3 months, I want to stop smoking and hope to soon, I REALLY want to stop smoking but I don’t know when I will, I want to stop smoking but haven’t thought about when, I think I should stop smoking but don’t really want to, I don’t want to stop smoking, or don’t know. MTSS was dichotomized into those having motivation to stop in a defined time frame (in the next month, or 3 months) versus otherwise.

#### E-cigarette Measures

##### E-cigarette Use at Baseline and Follow-up.

If respondents had heard of and tried an electronic cigarette, they were asked, “How often, if at all, do you currently use an electronic cigarette?”: daily, less than daily but at least once a week, less than weekly but at least once a month, less than monthly, not at all, or don’t know. Don’t know responses were coded as nonusers. For analyses that separated daily versus non-daily e-cigarette users, the variable was recoded as: daily use versus less than daily but at least once a week, less than weekly but at least once a month, or less than monthly.

##### E-cigarette Type at Follow-up.

Respondents who used e-cigarettes less than monthly or more often were asked what type of electronic cigarette equipment they currently use the most. Response categories included: (1) disposable electronic cigarette (non-rechargeable), (2) a commercial electronic cigarette kit which is refillable with pre-filled cartridges, (3) a commercial electronic cigarette kit which is refillable with liquids, or (4) a modular system (I use my own combination of separate devices: batteries, atomizers, etc.). E-cigarette type was dichotomized into: cigalike, categories (1) and (2) versus tank, categories (3) and (4). Because data on current e-cigarette brand/model used most often was also collected, brand and type were cross checked to ensure our categorization was correct. E-cigarette type was changed to match brand/model in 27 cases (5%) where brand and type did not match using the brand data.

##### E-cigarette Type and Frequency of Use at Follow-up.

A measure was derived from the questions on e-cigarette use and type of e-cigarette used at follow-up: no e-cigarette use, non-daily use of cigalike, daily use of cigalike, non-daily use of tank, daily use of tank.

### Analyses

SAS 9.3 was used for all analyses. An attrition analysis was conducted to examine differences between respondents who were eligible at baseline and respondents who were successfully followed-up with versus lost to attrition using Pearson chi-square analyses. For the main analyses, predictors of type of e-cigarette used at follow-up were first examined. Bivariate and multivariable logistic regression analyses were used to examine predictors of type of e-cigarette used at follow-up (tank vs. cigalike) among the 587 e-cigarette users at follow-up. Predictors examined included: gender, age, education, income, SUTS, and MTSS at baseline, and any use of e-cigarettes at baseline (less than monthly or more often vs. not at all). Any use of e-cigarettes at baseline was adjusted for because longer-term e-cigarette users (eg, those using at baseline and follow-up) may be more likely to use tanks.^1^ Additionally, because more frequent e-cigarette users may be more likely to use tanks, a sensitivity analysis was conducted by adding frequency of e-cigarette use at follow-up (daily vs. non-daily) into the model as a control variable to test whether the significant predictors of type of e-cigarette use changed. The association between e-cigarette type and frequency of e-cigarette use at follow-up and quitting smoking since baseline was then examined among the longitudinal sample (*N* = 1643). Smoking status at follow-up (ex-smoker vs. smoker) was set as the dependent variable in a multivariable logistic regression analysis adjusting for gender, age, education, income, SUTS, MTSS, and any e-cigarette use at baseline, with e-cigarette type and frequency of use at follow-up as the main independent variable of interest.

## Results

### Sample Characteristics and Attrition

Key demographic sample characteristics are shown in Table 1. Notable sample characteristics include the larger proportion of males (56.66%), and the high number of low income respondents (57.09% with annual household income under £30 000). Respondents who were followed up were significantly more likely at baseline to be male (*P* < .0001), from older age groups (*P* < .0001), have lower education (*P* = .0002), and have no motivation to quit in the next 3 months (MTSS; *P* = .0013); no differences were found by income (*P* = .1326), SUTS (0.1515), or e-cigarette use (*P* = .1318). Because e-cigarette type was only assessed at follow-up this prevented attrition analyses on this key variable. At follow-up, 64% reported no e-cigarette use, 27% used cigalikes, and 9% used tanks.

**Table 1.** Key Demographic Sample Characteristics (*N* = 1643)

Variable	*N*	%
Gender		
Male	931	56.66
Female	712	43.34
Age		
18–24	164	9.98
25–39	459	27.94
40–54	528	32.14
≥55	492	29.95
Education		
Low	571	34.75
Medium	641	39.01
High	431	26.23
Income		
Low	938	57.09
Medium	379	23.07
High	180	10.96
No answer	146	8.89

### Predictors of E-cigarette Product Type

Of the 587 e-cigarette users, 448 used cigalikes (76.32%) and 139 (23.68%) used tanks. Bivariate analyses showed that at follow-up the following groups were more likely to use tanks versus cigalikes: 40–54 year olds versus 18–24 year olds, and respondents with low versus moderate or high education. Gender, income, SUTS, MTSS, and e-cigarette use at baseline did not predict tank versus cigalike use at follow-up (Table 2). The multivariable analysis that adjusted for all variables that were examined in bivariate analyses produced similar results, with no changes in the comparisons that were significant at *P* < .05. A sensitivity analysis that added a control variable for frequency of e-cigarette use at follow-up did not lead to any changes in the baseline predictors of type of e-cigarette use at follow-up that were significant at *P* < .05. However, frequency of use at follow-up was associated with type of e-cigarette used at follow-up, such that, daily e-cigarette users at follow-up had 3.46 times greater odds of using tanks (35.94%) compared to non-daily users (17.72%) (95% CI =1.89 to 6.32, *P* < .0001).

**Table 2.** E-cigarette Users: Baseline Predictors of Using a Cigalike Versus Tank E-cigarette at Follow-up (*N* = 587)^a^

Variable			Bivariate				Multivariable			
	*N*	% using tank	*OR* ^b^	LCI	UCI	*P*	*OR*	LCI	UCI	*P*
Gender										
Male	309	22.33	1.00	1.00	1.00	ref	1.00	1.00	1.00	ref
Female	278	25.18	1.17	0.80	1.71	.4176	1.12	0.75	1.67	.5744
Age										
18–24	56	14.29	1.00	1.00	1.00	ref	1.00	1.00	1.00	ref
25–39	183	19.67	1.47	0.64	3.38	.3649	1.77	0.75	4.16	.1900
40–54	188	27.66	**2.29**	**1.02**	**5.18**	**.0455**	**2.41**	**1.05**	**5.52**	**.0378**
≥55	160	26.88	2.21	0.97	5.04	.0606	2.16	0.92	5.08	.0774
Education										
Low	187	33.69	1.00	1.00	1.00	ref	1.00	1.00	1.00	ref
Moderate	233	22.32	**0.57**	**0.37**	**0.87**	**.0098**	**0.57**	**0.37**	**0.88**	**.0118**
High	167	14.37	**0.33**	**0.20**	**0.56**	**<.0001**	**0.36**	**0.21**	**0.63**	**.0003**
Income										
Low	330	25.15	1.00	1.00	1.00	ref	1.00	1.00	1.00	ref
Moderate	144	24.31	0.96	0.61	1.51	.8447	1.24	0.76	2.01	.3849
High	72	15.28	0.54	0.27	1.07	.0764	0.83	0.40	1.72	.6160
No answer	41	24.39	0.96	0.45	2.04	.9155	0.93	0.43	2.02	.8496
Strength of urges to smoke^c^										
0—no urge	21	28.57	1.08	0.90	1.30	.4192	1.05	0.87	1.28	.602
1	61	26.23								
2	267	22.10								
3	172	21.51								
4	45	24.44								
5—strong urges	21	47.62								
Motivation to stop smoking										
No motivation to stop in next 3 months	427	25.53	1.00	1.00	1.00	ref	1.00	1.00	1.00	ref
Motivation to stop in next 3 months	160	18.75	0.67	0.43	1.06	.0868	0.75	0.47	1.21	.2343
Any e-cigarette use										
No e-cigarette use	328	25.61	1.00	1.00	1.00	ref	1.00	1.00	1.00	ref
E-cigarette use	259	21.24	0.78	0.53	1.15	.2163	0.81	0.54	1.22	.3052

LCI = lower 95% confidence interval; UCI = upper 95% confidence interval.

^a^Bold indicates significant at *P* < .05.

^b^ *OR* = odds ratio.

^c^Continuous variable.

### E-cigarette Product Type, Frequency of Use, and Smoking Cessation

Multivariable analyses showed that quitting smoking since baseline was associated with frequency and type of e-cigarette used at follow-up (Table 3). Adjusting for other factors that predict quitting, compared to respondents who reported no e-cigarette use at follow-up: respondents who used cigalikes non-daily were less likely to have quit, respondents who used cigalikes daily were no more or less likely to have quit, respondents who used tanks non-daily were no more or less likely to have quit, and respondents who used tanks daily were more likely to have quit. Baseline factors that predicted quitting included income, MTSS, and SUTS. Smokers who had higher incomes versus lower incomes, experienced weaker urges to smoke, and were motivated to stop smoking in the next 3 months at baseline were more likely to have quit at follow-up.

**Table 3.** E-cigarette Use, Product Type, and Quit Smoking at Follow-up, *N* = 1643^a^

Variable	*N*	% Quit smoking	*OR* ^b^	LCI	UCI	*P*
**Baseline**						
Gender						
Male	931	11.39	1.00	1.00	1.00	ref
Female	712	12.78	1.22	0.89	1.67	.2167
Age						
18–24	164	12.20	1.00	1.00	1.00	ref
25–39	459	15.03	1.33	0.76	2.33	.3171
40–54	528	11.55	0.97	0.55	1.69	.907
≥55	492	9.55	0.91	0.51	1.62	.7434
Education						
Low	571	11.73	1.00	1.00	1.00	ref
Medium	641	12.48	1.04	0.73	1.50	.8228
High	431	11.60	0.82	0.54	1.26	.3633
Income						
Low	938	10.23	1.00	1.00	1.00	ref
Medium	379	13.72	1.21	0.83	1.78	.319
High	**180**	**16.11**	**1.63**	**1.00**	**2.66**	**.0483**
No answer	146	13.70	1.40	0.82	2.39	.2201
Motivation to stop smoking						
No motivation to stop in next 3 months	1288	9.7	1.00	1.00	1.00	ref
**Motivation to stop in next 3 months**	**355**	**20.28**	**2.54**	**1.81**	**3.56**	**<.0001**
**Strength of urges to smoke^c^**						
**0—no urge**	**126**	**23.81**	**0.77**	**0.66**	**0.89**	**.0004**
1	212	16.04				
2	764	10.47				
3	387	9.82				
4	105	8.57				
5—strong urges	49	12.24				
Any e-cigarette use at baseline						
No e-cigarette use	1295	12.74	1.00	1.00	1.00	ref
E-cigarette use	348	9.20	0.83	0.52	1.30	.4067
**Follow-up**						
**E-cigarette type and frequency of use at follow-up**						
No e-cigarette use	1056	13.45	1.00	1.00	1.00	ref
**Non-daily cigalike**	**325**	**5.23**	**0.35**	**0.20**	**0.60**	**.0002**
Daily cigalike	123	10.57	0.74	0.39	1.42	.3644
Non-daily tank	70	8.57	0.70	0.29	1.68	.4216
**Daily tank**	**69**	**27.54**	**2.69**	**1.48**	**4.89**	**.0012**

LCI = lower 95% confidence interval; UCI = upper 95% confidence interval.

^a^Bold indicates significant at *P* < .05.

^b^ *OR* = odds ratio.

^c^Continuous variable.

## Discussion

In a sample of smokers at baseline, predictors of using tanks versus cigalikes at a 1-year follow-up included low versus moderate and high education, and middle (40–54) versus younger age (18–24). Daily e-cigarette users at follow up were also more likely to use tanks than non-daily users. This study additionally found that when adjusting for baseline factors that predict quitting, there was a cross-sectional association between type and frequency of e-cigarette used, and quitting smoking at follow-up. Compared with respondents not using e-cigarettes at follow-up, respondents who were using a tank e-cigarette daily were more likely to have quit. Respondents using tanks non-daily or a cigalike daily were no more or less likely to have quit, while those using cigalikes non-daily were less likely to have quit.

This is the first longitudinal study drawn from a general population sample to examine the association between quitting smoking and type and frequency of e-cigarette use. Because different associations were found depending on type and frequency of e-cigarette use, this study suggests that it is important to consider type and frequency of e-cigarette use when examining the association between e-cigarette use and quitting smoking.

The findings of this study are similar to previous studies that have suggested that people using tanks are more likely to be ex-smokers.^25,38^ Additionally, the findings that those who use e-cigarettes more frequently (daily vs. non-daily) are more likely to quit, are similar to findings by Biener and Hargraves^30^ who found that it was only more intensive e-cigarette use that predicted quitting compared to no use, and Brose et al.^29^ who found that only daily e-cigarette users were more likely to have reduced their cigarette consumption and made quit attempts. It is also worth noting that the association between more intensive e-cigarette use and quitting is similar to findings that suggest that greater adherence to nicotine replacement therapy may predict quit success.^39,40^ Additionally, similar to previous studies in other samples of English smokers, motivation to stop smoking, weaker urges to smoke (a measure of nicotine dependence), and higher income (an indicator of socioeconomic status), predicted quitting in this study.^36,37,41^ The fact that these variables still predicted quitting after adjusting for frequency and type of e-cigarette use suggest that e-cigarettes did not close the gap in inequities in quitting in this sample. Further research should investigate the impact of e-cigarettes on quitting for groups that have been found to be less likely to successfully quit (more nicotine dependent, and lower socioeconomic status).^41,42^

These findings, showing an association between e-cigarette type, frequency of use, and quitting, should be considered in the context of the e-cigarette regulatory environment. For example, they may be particularly relevant due to calls by the tobacco industry for stringent regulations for tanks in the United States (along with the fact that, to the authors’ knowledge, that most tobacco companies sell only cigalikes), and restrictions on tank type e-cigarettes in some healthcare settings.^43–45^

This study has several limitations. A low follow-up rate of 43.3% and differential loss to follow-up mean that the findings may not apply to all groups. Because e-cigarette type was only assessed at follow-up, we were unable to ascertain if there was differential loss to follow-up by e-cigarette type and frequency of use. Additionally, although efforts were made to ensure the representativeness of the sample at baseline, this was not a nationally representative survey, and caution should be taken when applying the results to the general population, particularly given the low follow-up rate. The data were also self-reported. Additionally, because e-cigarette type and frequency of use was measured at follow-up conclusions cannot be made about whether use is predictive of later cessation because the direction of causation might be reversed. For example, it could be that there is no association between e-cigarette type and quitting, and instead that established e-cigarette use, using a tank model, and quitting smoking are correlated. However, the analyses were adjusted for a variety of sociodemographic and smoker characteristics at baseline. Whether the e-cigarette was used with nicotine was not measured, nor was duration of use, however, we did adjust for use of e-cigarettes at baseline.

Despite limitations, this study has several strengths, a sample that was drawn from the general population in Britain, adjustment for factors that predict quitting at baseline in models predicting quitting, and detailed information on type of e-cigarette used that was cross checked with brand and model. Additionally, as previously stated, several variables that have been previously demonstrated to predict quitting also predicted quitting in this study.

Future research using longitudinal studies and randomized control trials should further investigate the association between e-cigarette use and smoking behavior, that is, whether they promote or deter smoking cessation. Such studies should consider frequency of use, type of e-cigarette, whether used during a quit attempt, after quitting, for temporary abstinence/cutting down, duration of use, and nicotine content. Future research should also consider the role of user preferences. Indeed, the current study demonstrates that the users of tanks versus cigalikes differ. It will also be important to understand why type of e-cigarette used is associated with smoking cessation; for example, effectiveness for smoking cessation may depend on differences in nicotine delivery, relieving urges to smoke, user satisfaction, psycho-social influences (eg, norms), bio-behavioral feedback, and/or marketing, including promotion and price.

## Funding

All authors are members of the UK Centre for Tobacco & Alcohol Studies, a UK Clinical Research Collaboration Public Health Research: Centre of Excellence whose work is supported by funding from the Medical Research Council, British Heart Foundation, Cancer Research UK, Economic and Social Research Council, and the National Institute for Health Research under the auspices of the UK Clinical Research Collaboration is gratefully acknowledged (MR/K023195/1). JB’s post is funded by a fellowship from the UK Society for the Study of Addiction. The funders played no role in the study design, collection, analysis, and interpretation of the data, in the writing of the manuscript and in the decision to submit this manuscript for publication.

## Declaration of Interests

*JB has received an unrestricted research grant from Pfizer related to the surveillance of smoking cessation trends. LSB, SCH, DR, and AM have no conflicting interests to declare.*
